# Quantitative Morphometry for Osteochondral Tissues Using Second Harmonic Generation Microscopy and Image Texture Information

**DOI:** 10.1038/s41598-018-21005-9

**Published:** 2018-02-12

**Authors:** Takashi Saitou, Hiroshi Kiyomatsu, Takeshi Imamura

**Affiliations:** 1 Department of Molecular Medicine for Pathogenesis, Graduate School of Medicine, Ehime University, Shitsukawa, Toon-city, Ehime 791-0295 Japan; 2 Translational Research Center, Ehime University Hospital, Shitsukawa, Toon-city, Ehime 791-0295 Japan; 3 Division of Bio-Imaging, Proteo-Science Center (PROS), Ehime University, Shitsukawa, Toon-city, Ehime 791-0295 Japan; 4 Department of Orthopedic Surgery, Graduate School of Medicine, Ehime University, Shitsukawa, Toon-city, Ehime 791-0295 Japan

## Abstract

Osteoarthritis (OA) is a chronic joint disorder involving degeneration of articular cartilage and subchondral bone in joints. We previously established a second harmonic generation (SHG) imaging technique for evaluating degenerative changes to articular cartilage in an OA mouse model. SHG imaging, an optical label-free technique, enabled observation of collagen fibrils, and characterized critical changes in the collagenous patterns of the joints. However, it still remains to be determined how morphological changes in the organization of tissue collagen fibrils should be quantified. In this study, we addressed this issue by employing an approach based on texture analysis. Image texture analysis using the gray level co-occurrence matrix was explored to extract image features. We investigated an image patch-based strategy, in which texture features were extracted on individual patches derived from original images to capture local structural patterns in them. We verified that this analysis enables discrimination of cartilaginous and osseous tissues in mouse joints. Moreover, we applied this method to OA cartilage pathology assessment, and observed improvements in the performance results compared with those obtained using an existing feature descriptor. The proposed approach can be applied to a wide range of conditions associated with collagen remodeling and diseases of cartilage and bone.

## Introduction

Articular cartilage is a specialized avascular connective tissue that covers the ends of long bones. It serves to distribute loads on bones, absorb impacts, and provide smooth articulation and protection for the underlying bone^1^. Articular cartilage is composed of extracellular matrix (ECM) whose main components are a dense network of type II collagen fibers, hydrated proteoglycans, a large amount of interstitial water, and a sparsely distributed population of chondrocytes. Chondrocytes are cells embedded within the ECM that are responsible for its synthesis and maintenance. The modification of the composition and structure of the ECM can cause pathological states of cartilage. Osteoarthritis (OA), the most common form of arthritis, is initiated by the deterioration of chondrocytes, loss of proteoglycans, and modification of the collagen network, which leads to the degradation of articular cartilage and subchondral bone^2–4^. Imaging methods such as X-rays, magnetic resonance imaging (MRI) and computed tomography (CT) have been used clinically to evaluate cartilage degeneration in OA. X-rays and CT provide useful diagnostic information by detecting morphological changes in bone and calcified tissues, but these techniques are limited to detecting the late stages of OA progression. MRI can be used to detect degenerative changes in cartilage; however, due to limited resolution, it is difficult to detect early changes in articular cartilage^5^. Therefore, it is highly desirable to develop a novel diagnostic method that can allow more sensitive cartilage assessment to evaluate disease progression.

Second harmonic generation (SHG) microscopy has become a vital tool for visualizing extracellular collagen in living tissues directly without tissue staining. It is a nonlinear and coherent optical process where two excitation photons are effectively combined in an optically nonlinear medium, to create an energy-doubled photon at half the wavelength of the excitation source. Molecules possessing non-centrosymmetric structures emit a strong SHG signal. Due to its nonlinearity, this technique allows highly localized optical excitation, resulting in high resolution imaging. In living tissues, since the SHG signal comes specifically from collagen molecules, SHG has been used extensively for label-free imaging of fibrillar organization, and applied to the assessment of various diseases^6–11^. Approaches using the SHG imaging technique to assess degeneration of the cartilage matrix and its pathological states have been reported^12–16^. Although morphological studies of articular cartilage using SHG microscopy are now becoming popular, the development of quantitative methods which allow detection and automatic classification of disease states is not well established.

In this study, we addressed this issue by combining the technique of image texture analysis^17–20^ with SHG imaging. We employed a statistical texture analysis method using the gray-level co-occurrence matrix (GLCM)^21^, which has attracted attention to applications in MRI^22–24^, CT images^25^, ultrasound images^26^, histopathological images^27^, and cellular level images^28,29^. By using this computational method, we developed a patch-based texture feature extraction scheme, in which the whole image is divided into pieces of cropped images and texture features are obtained in each patch for quantitative characterization of SHG images of the cartilaginous and osseous tissues. The image patch size was chosen to include particular structures observed in the tissues, the sparsely distributed chondrocytes, and the surrounding ECM. We showed how this analytical method discriminates the tissue collagen types using images from tissue sections. Furthermore, based on the extracted texture features, we explored the use of a generic image classification method, Bag of Features (BoF)^30^, in order to evaluate the discrimination potential. The BoF method is based on a collection of independent units, called visual words, which correspond to quantized local features extracted from image patches. Due to the simplicity of its implementation and the high performance of classification, this method is well established in the field of generic image categorization^31^. BoF has been applied to the classification of histopathological images^32,33^, and two photon excitation fluorescence images^34,35^. We have demonstrated the utility of texture information from SHG images and Bag of Features (BoF)-based image classification analysis. Moreover, we applied this established method to OA cartilage pathology assessment. Our study is aimed at developing a quantitative collagen feature descriptor in osteochondral tissues based on texture information. The proposed texture extraction technique and the BoF image classification strategy would provide a potential diagnostic tool for OA assessment of collagenous SHG images.

## Results

### SHG imaging captures morphological distinctions between cartilage and bone tissues

**Figure 1:**
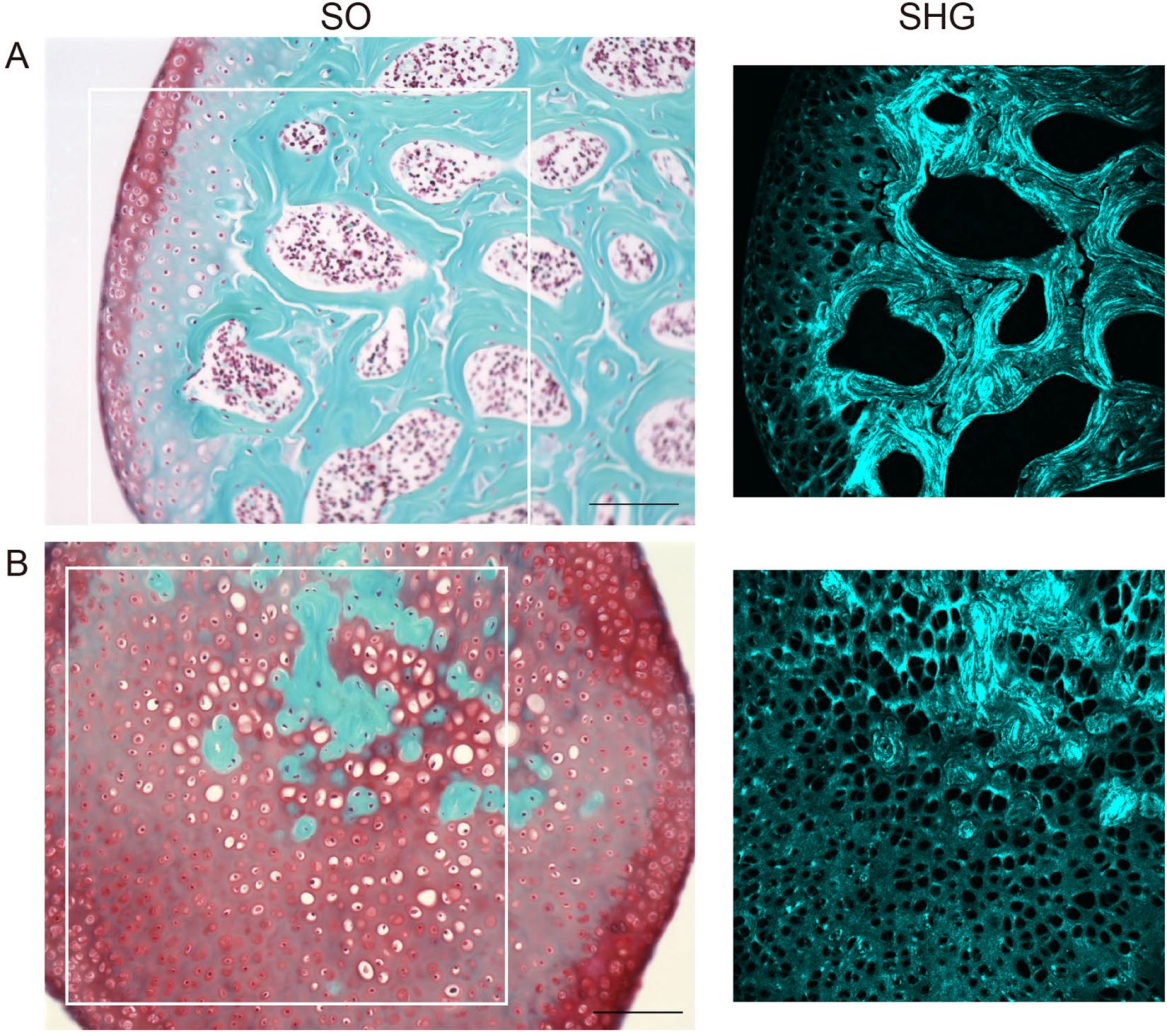
Histological and SHG images of frontal sections from the normal medial condyle in the distal femur. Left: Bright-field images of the SO-stained sections. Right: SHG images corresponding to the white frames in the SO (left) images. These are representative images that captured the subchondral bone (**A**) and cartilage (**B**). Scale bar: 100 μm.

In order to investigate how SHG imaging characterizes the morphological differences between cartilage and bone tissues of mouse knee joints, we first performed SHG imaging and histological analyses of tissue sections by using a multi-photon excitation microscope. Frontal sections were obtained from the normal medial condyle of the distal femur for a total of four mice. The SHG images were captured prior to histological staining of the tissue sections, and histological examination was performed using Safranin O Fast Green (SO) staining of the sections (Fig. 1). The articular cartilage has a smooth surface, and in this peripheral region, the cartilage was well-stained in red on the SO images (Fig. 1A,B SO). This indicated a normal structure for the articular cartilage in which chondrocytes and chondrocytic lacunae surrounded by the ECM were discernible. The subchondral bone was stained in blue within the interior of the condyle (Fig. 1A,B SO). The SHG signal can be detected in the ECM of the surface layer of articular cartilage, whereas the chondrocytic lacunae did not show any signals, indicating that the hyaline cartilage was selectively imaged with SHG signals (Fig. 1A,B SHG). On the other hand, the SHG signal emitted by the subchondral bone ECM was stronger than that from the cartilage matrix, exhibiting a fibrous pattern of osseous collagen structures. Therefore, the SHG images enabled us to differentiate the cartilage and subchondral bone tissues morphologically in the knee joints.

**Figure 2:**
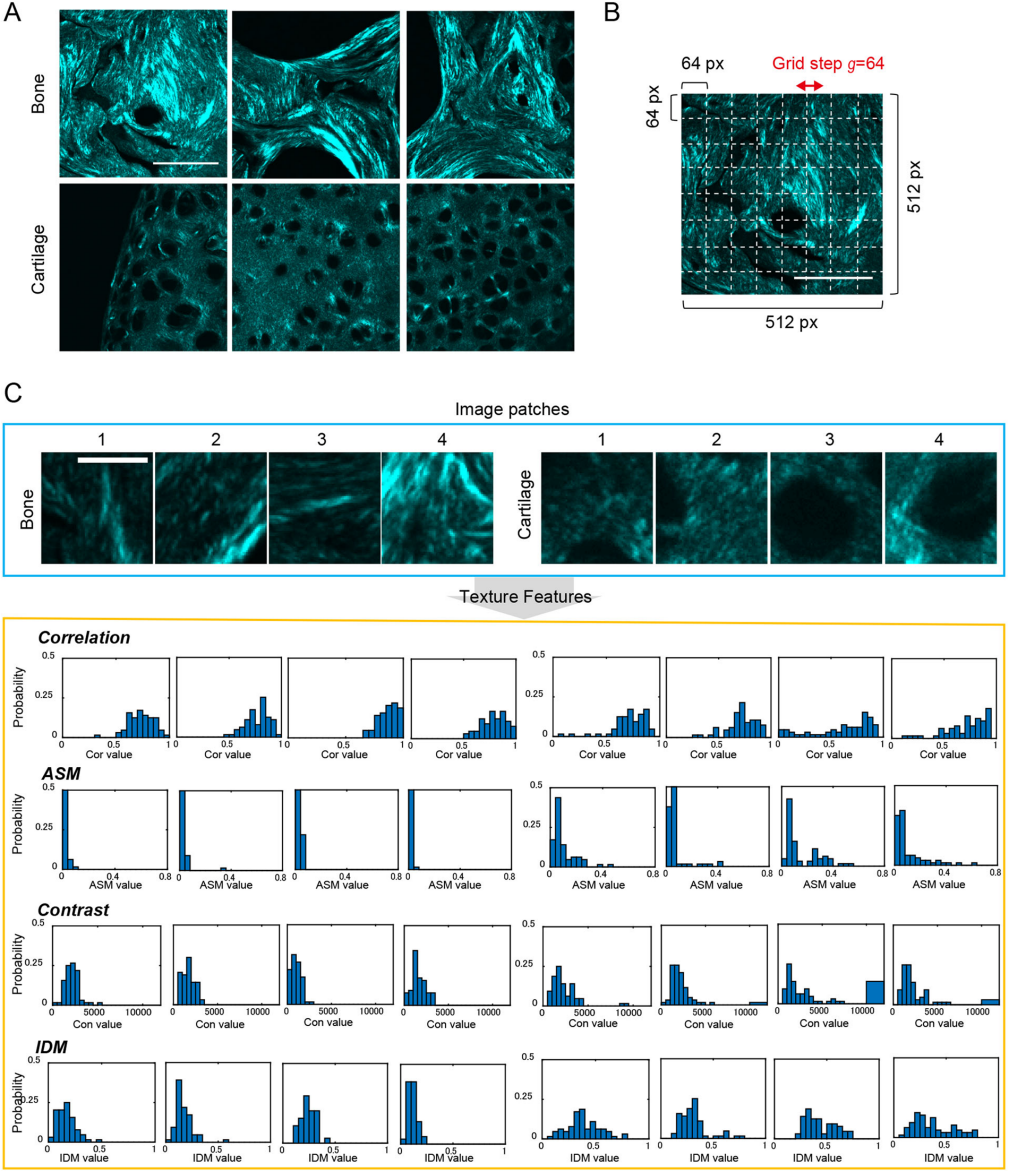
Scheme for texture feature extraction from SHG images. (**A**) Representative images of the subchondral bone (upper) and articular cartilage (lower). Scale bar: 50 μm. (**B**) Image patches to be subjected to feature vector calculations are selected according to the regular square grid (white dashed lines). This grid is defined by the parameters of the grid step and the image patch size, where the grid step is the distance between neighboring patches. (**C**) Next, the patches are subjected to the texture parameter calculation using the GLCM. Correlation, ASM, contrast, and IDM corresponding to the images presented in (**B**) are shown. Scale bar: 10 μm. Cor value: Corralation value. Con value: Contrast value.

On the basis of these observations, we performed image categorization in order to conduct quantitative image analysis using texture information. As described in detail in the Methods section, the SHG images were examined based on the SO staining results, and the 512 × 512 pixel size sub-images were obtained manually from the original images (2048 × 2048 pixels) in such a way that each cropped imaged is confined almost exclusively to either the cartilage or subchondral bone regions (Fig. 2A). The images obtained were therefore categorized as either ‘Bone’ or ‘Cartilage’ images, before being subjected to analysis.

### Texture features quantify morphological characteristics of cartilage and bone tissues

By using SHG image sets categorized as either ‘Bone’ or ‘Cartilage’ (Fig. 2A), we next considered how to extract texture features reflecting the morphological characteristics of the SHG images. In order to do this, we employed a patch-based feature extraction scheme to capture the local morphologies of the cartilaginous and the osseous tissues from the images. The whole image was divided into pieces of cropped images and texture features were obtained from each patch. The image patch-based strategy is compatible with the BoF image classification approach, which is based on a collection of local image patches. The image patches were taken as 64 × 64 pixel images corresponding to a 16 × 16 μm^2^ area, large enough to capture the local structures, since the distance between the neighboring chondrocytic lacunae was estimated at 10–20 μm. The image patches hence include few lacunae, and thus these may be typical size for characterizing morphology (Fig. 2B). Features were chosen to locate at the fixed regular grid locations, defined by the grid step *g*, which was either 32 or 64 pixels (Fig. 2B).

Image texture analysis is a mathematical method for enumerating complex visual patterns that reflects homogeneity, density, roughness, regularity, frequency, and randomness, etc. Approaches to image texture analysis are categorized into the following ways: structural, statistical, model-based, and transform^17–19^. In this study, we employed a statistical texture analysis method using the gray-level co-occurrence matrix (GLCM)^21^. The cartilaginous and osseous SHG images possess a large amount of textural information without any particular formed patterns. Therefore, the statistical approach seems to be suitable for our purpose. The GLCM, denoted as *p*(*i*, *j*), represents the probability of occurrence of pixel pairs with gray levels *i* and *j*, which are separated from each other by a distance *d* along a given direction *θ* (Supplementary Fig. 1A). In this study, the GLCM was calculated in four orientations, namely horizontal, vertical, and two diagonals (*θ* = 0°, 45°, 90°, or 135°). From this matrix, several parameters can be calculated. Here, we measured texture parameters of correlation, angular second momentum (ASM), contrast, inverse difference moment (IDM), entropy, sum entropy, sum average, and sum variance. In order to obtain these parameters of the image patches, the following calculation strategy was adapted. We first selected regions of interest (ROIs) each composed of 8 × 8 blocks of 8 × 8 pixel images divided from an image patch. The GLCM calculation was done individually on each ROIs and subsequently the texture parameters were computed on each ROI (Supplementary Fig. 1B). The texture parameters, correlation, and ASM, calculated from the patches shown in Fig. 2C were plotted in Supplementary Figure 2. Overall, ASM values in Bone patches were lower than those in Cartilage ones, indicating greater homogeneity for collagen structures in cartilaginous tissues. Within Bone patches, ASM values at which fibrous collagens exist were relatively lower, while within Cartilage patches, lacunae showed relatively higher ASM values compared with the surrounding ECM. Meanwhile, in the correlation maps, relatively higher values were detected where SHG signals are observed, reflecting the high linearity of collagenous structures. These data demonstrated that the texture parameters can be used to provide the quantitative morphological features of image patches for discriminating cartilaginous and osseous SHG signals. Next, we constructed feature vectors for the image patches by calculating the histogram of texture parameters within the image patch. Results derived from randomly selected image patches are shown in Fig. 2C. The distribution of correlation in Bone patches was concentrated in the latter half (0.5–1), while those in Cartilage patches are also concentrated in the same range, but these patches have values within a lower range, indicating the presence of higher linear structures in Bone patches. From the ASM histogram, it is clear that the distribution in Bone patches was located in a lower range (<0.2), however, that in Cartilage patches extends to a higher range (up to 0.6). This suggested that higher homogeneous images are included in Cartilage, possibly reflecting the existence of lacunae which appear as dark holes with little variance in gray levels. The parameters for contrast and IDM also exhibited different behaviors between Cartilage and Bone patches. Higher contrast values were observed more in Cartilage than in Bone, and IDM was distributed about a higher range in Cartilage. Furthermore, entropy and sum entropy showed different behaviors, while sum average and sum variance showed no apparent differences between Cartilage and Bone images (Supplementary Figs S3 and S4). Based on these results, we concluded that the texture parameters statistically summarized to histograms contained discriminant information for analyzing Bone and Cartilage image sets.

### Texture feature-based machine learning for image classification

**Figure 3:**
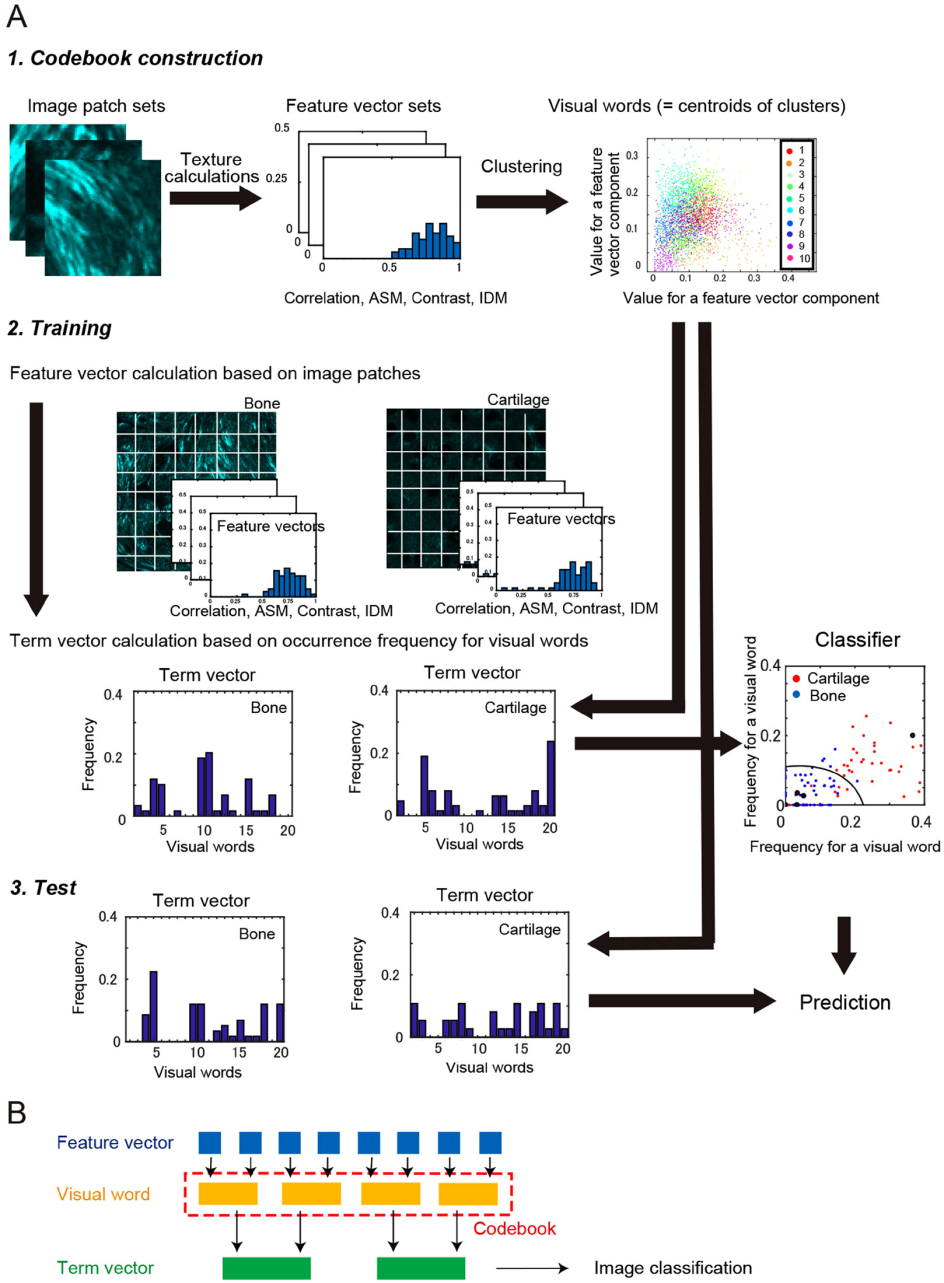
Schematic description of the BoF framework. (**A**) BoF comprises three steps, namely codebook construction, training, and test. In the codebook construction step, image patches are extracted and feature vectors calculated using the GLCM method. These vectors are used to create visual words by using the *k*-means clustering method. In the training step, an image is divided into patches that are subjected to feature vector calculation. A histogram of visual words occurrence (term vector) is subsequently calculated for each image. Based on these data, the classifier is constructed. In the test step, image prediction is performed based on the SVM (or multi-class ECOC) classifier. (**B**) Hierarchical relationship between terminologies: feature vector, visual word, codebook, and term vector. Beginning with feature vectors derived from image patches, visual words are created using a *k*-means clustering method. Codebook is a collection of visual words. Term vectors are histograms of visual word occurrence within an image.

**Figure 4:**
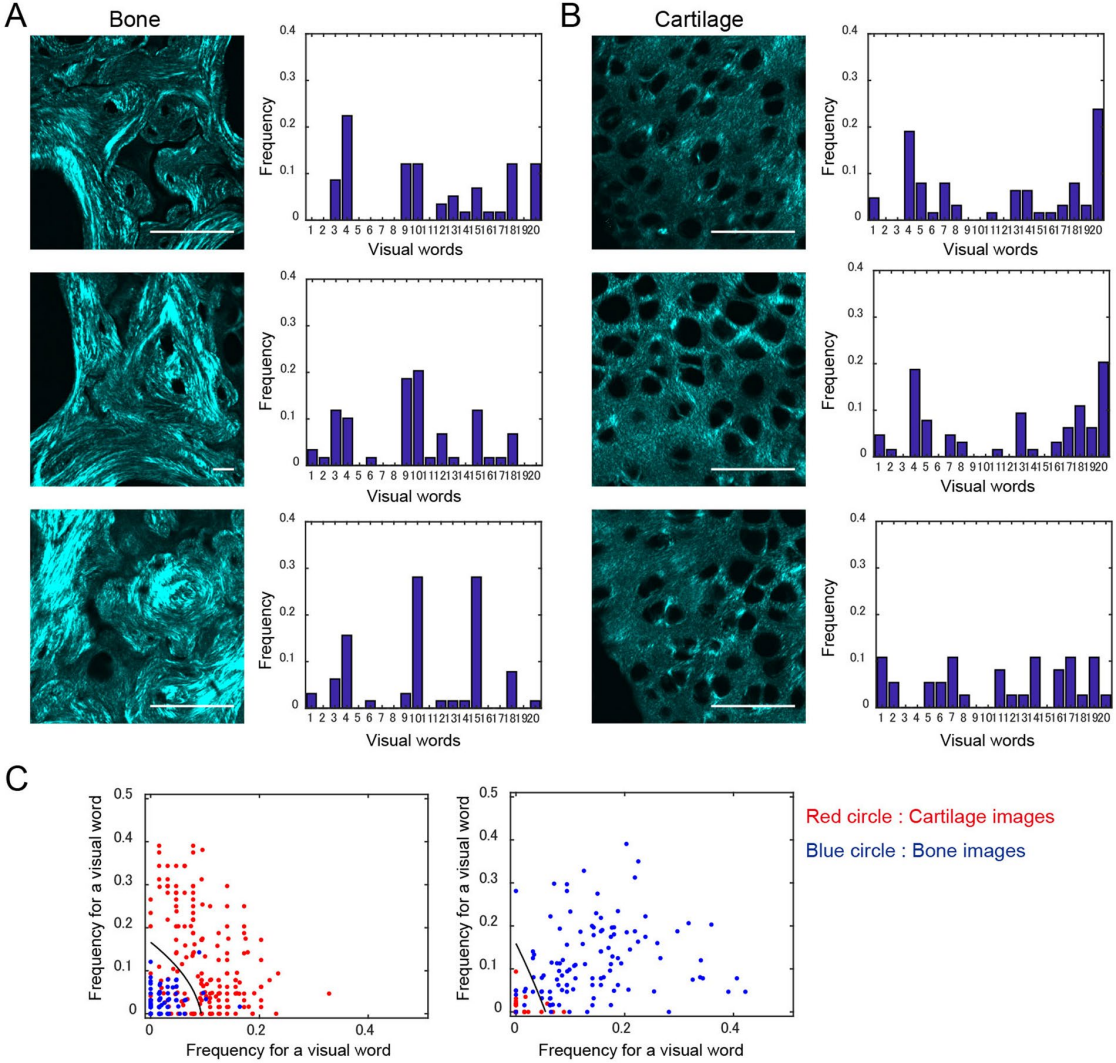
Term vector calculation and training with images. Representative SHG images and term vectors (visual word occurrence histogram) are shown for the subchondral bone (**A**) and cartilage tissues (**B**). The results exhibited are *k* = 20 case. (**C**) Results of the training data set. The classifier was constructed by the SVM machine learning method. Scale bar: 50 μm.

In order to determine the discrimination ability of texture parameters, we employed the BoF framework, a machine learning approach for image classification. The BoF method is a popular visual classification method used in a variety of applications^31^. In brief, this method is based on an unordered collection of quantized image descriptors derived from local image patches. An image is represented as a histogram of the number of occurrences for particular patterns of image patches, called visual words. The BoF framework adjusted to make use of the texture information is illustrated in Fig. 3A. In order to make a terminological distinction, the hierarchical relationship is illustrated in Fig. 3B. The framework was comprised of three parts, namely codebook construction, training, and testing. In the codebook construction step, patches were extracted from categorized images, and texture parameters were calculated for each patch using the GLCM method. We assigned a feature vector for an image patch by integrating the texture parameters calculated in the previous section. Thus, the resulting one was a vector with a dimension of 352 elements. The feature vectors collected from the image patch sets were used to create visual words by using the *k*-means clustering method. During the training step, in order to represent image properties numerically, we created a histogram of visual word occurrences (term vector) for each image. In order to do so, the image was divided into patches which were subjected to feature vector calculations. The term vector was subsequently calculated based on *k*-clustered visual words (Fig. 4A,B).

Next, based on these data, a classifier was constructed (Fig. 4C). In the test step, using the classifier and visual words, image prediction was performed by assigning a term vector and running the vector through a classifier that uses the term vectors of the training images. For details, see the Methods section. To compare existing feature extraction methods, we used the speeded-up robust feature (SURF) which is a popular gradient-based feature description technique^36^. The texture-BoF algorithm depends on three variable parameters, namely the image patch size, the grid step *g*, and codebook size, *k*. The image patch size is fixed at 64 × 64 pixels for reasons described above. The grid step, which is the distance in pixels between the nearest neighbor patches, was set at either *g* = 32 or 64. The codebook size, which is the number of visual words, was taken as either *k* = 20, 50, or 100. We investigated parameter sensitivities for the image classification by varying these parameter values.

We showed the computed term vectors for representative Cartilage and Bone images in the case where *k* = 20 (Fig. 4,B). Comparing the term vectors for Cartilage and Bone images demonstrates the difference in distribution patterns. The differences can be recognized at a *k* value of 9 or 10. Figure 4C showed classification results for the training data set, and these demonstrated a good separation of the two categorized images in the space of the specific *k* values.

Next, we evaluated the classification performance of the texture- and the SURF-BoF framework for the SHG image sets. The prediction by the trained classifier was represented in a structure known as a confusion matrix (Table 1 and Supplementary Table 2). The results shown are averaged values for four consecutive runs of the classification test designated in Supplementary Table 1. Overall, the results showed higher accuracy for the texture-BoF than that for the SURF-BoF. For example, in the case that *k* = 20, the texture-BoF framework achieved 97.5% accuracy for Cartilage images and 94.4% accuracy for Bone images. On the other hand, within the SURF-BoF framework, the accuracies were 75.5% and 78.5% for Cartilage and Bone images, respectively. When we changed the codebook size, the accuracy values were affected; however, the tendency for texture-BoF to have better classification accuracy did not change. We next changed the parameter of the grid step size, *g* = 32, indicating an increased number of patches extracted from an image. The results showed that the classification becomes more accurate than the case for *g* = 64 both for the texture- and SURF-BoF methods. Finally, it is noteworthy that the texture-BoF classification results are independent of the codebook size, while the accuracy values in the SURF-BoF decreased as the codebook size decreased. This result indicated that the texture-BoF framework does not need to create a larger codebook size.

**Table 1:** Results of BoF classification tests performed on SHG images of tissue sections.

	Cartilage	Bone
*k* = 20, *g* = 64		
Cartilage	0.975	0.025
Bone	0.062	0.944
*k* = 50, *g* = 64		
Cartilage	0.998	0.002
Bone	0.057	0.943
*k* = 100, *g* = 64		
Cartilage	0.994	0.006
Bone	0.057	0.943
*k* = 50, *g* = 32		
Cartilage	0.993	0.007
Bone	0.062	0.954

The probability of the confusion matrix is shown. Texture BoF method for *k* = 20, 50, or 100 and *g* = 32, 64 are shown.

### Applying the texture-BoF algorithm to OA cartilage pathology discrimination

**Figure 5:**
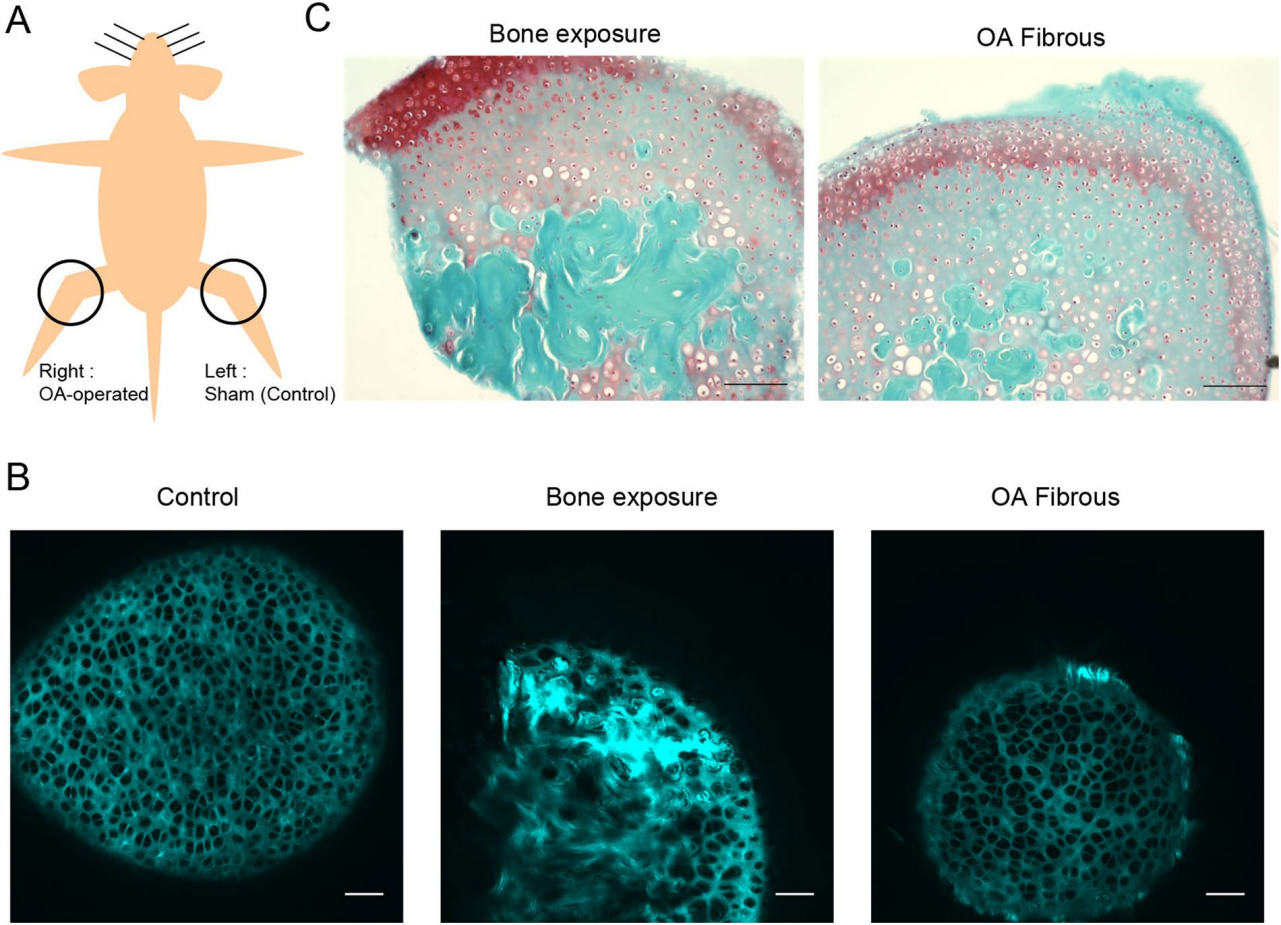
Applying the BoF texture analysis to the classification of SHG images of the OA model mice. (**A**) Construction of the OA mouse model. The right knee is the OA-operated side while the left knee is the sham-operated control side. Scale bar: 100 μm. (**B**) Representative SHG images of normal cartilage from the control side (left), and degenerative cartilage from the OA side, bone exposure (middle), and hyperplasia of fibrous cartilage (right). Scale bar: 50 μm. (**C**) Histological tissue sections from the OA-side. Typical images showing cartilage degeneration, bone exposure (left) and fibrous cartilage (right) are shown.

In order to apply the texture-BoF algorithm to discrimination of the OA pathological state, we used OA model mice in which instability in the knee joints was induced surgically^37^. A total of 12 mice were subjected to surgery. For each mouse, the right knee was OA-operated and the left knee was sham-operated (Fig. 5A). Eight weeks after surgery, the femurs were excised, and *ex vivo* imaging with SHG of the medial condyle of the distal femur was performed. Representative SHG images of the cartilage tissue from the OA model were shown in Fig. 5B. An image from the control side showed a typical structure of the articular cartilage consisting of the chondrocytic lacunae and ECM (Fig. 5B, left). In contrast to the control side, an OA side image exhibited heterogeneous signal patterns and intensity distributions. SHG signals are strong in part, whereas the surface of the articular cartilage was uneven (Fig. 5B, middle). The signal patterns resembled those observed in sectioned SHG images of the subchondral bone, suggesting defects in the articular cartilage and exposure of the subchondral bone. Furthermore, on some of the OA mice, fibrous collagenous patterns on the surface of the cartilage were observed (Fig. 5B, right), indicating hyperplasia of the fibrous cartilage. These observations reflected typical characteristics of cartilage degeneration in the OA model^14^.

Following the *ex vivo* image analysis, histological sections were subjected to SO staining. We confirmed the exposure of the subchondral bone (Fig. 5C, left) and hyperplasia of the fibrous cartilage by SO staining tissue sections (Fig. 5C, right). Based on the results from SHG and histological analyses, we categorized the SHG images into three distinct classes, namely ‘Hyaline’, where the hyaline cartilage was dominant, ‘Fibrous’, where fibrous cartilage in the cartilage surface was observed, and ‘Bone’, where exposure of the subchondral bone was detected, so as to determine the utility of the texture-BoF algorithm for discriminating OA pathology (Supplementary Table 3).

As in the test for the sectional SHG images, we evaluated the prediction accuracy of the texture- and SURF-BoF frameworks. The results were summarized in the confusion matrices (Table 2 and Supplementary Table 4). Overall, the prediction tests for the texture-BoF were more accurate than those for the SURF-BoF, similar to the results demonstrated in the tests for tissue sections. The texture-BoF framework achieved greater than 90% accuracy for Hyaline and Bone image classifications, although the accuracy of Fibrous images was not as high, showing only 60–70% accuracy. However, since the accuracy in the SURF-BoF dropped to 28–42% for Fibrous images, the texture-BoF showed better performance. The codebook size independence in classification accuracy was also true in this case. When the grid step size was changed to *g* = 32, the results showed that the classification performance was higher, greater than 70% accuracy for Fibrous images.

**Table 2:** Results of BoF classification tests performed on SHG images of the OA model.

	Hyaline	Fibrous	Bone
*k* = 20, *g* = 64			
Hyaline	0.930	0.050	0.021
Fibrous	0.297	0.656	0.048
Bone	0.079	0.021	0.900
*k* = 50, *g* = 64			
Hyaline	0.975	0.013	0.012
Fibrous	0.322	0.589	0.090
Bone	0.044	0.019	0.938
*k* = 100, *g* = 64			
Hyaline	0.982	0.013	0.005
Fibrous	0.307	0.591	0.102
Bone	0.039	0.051	0.910
*k* = 50, *g* = 32			
Hyaline	0.973	0.021	0.006
Fibrous	0.163	0.715	0.122
Bone	0.023	0.030	0.947

The probability of the confusion matrix is shown. Texture BoF method for *k* = 20, 50, or 100 and *g* = 32, 64 are shown.

## Discussion

In order to establish quantitative morphometrics for osteochondral tissues, which are aimed at assessing diseases derived from collagenous disorganization, we have employed a combined approach involving both SHG imaging and image texture analysis, and demonstrated a methodology for performing the texture feature extraction from the tissue SHG images. In quantifying the collagen fibril morphology based on texture features, we developed an image patch-based analysis and evaluated the potential utility of this method for classifying images derived from both sectioned and intact cartilaginous and osseous tissues as either normal or diseased based on a mouse OA model by using the BoF based machine learning framework. The combined texture and BoF classification framework proposed in this study provides a promising approach, and thus holds significant potential with respect to the assessment problem for cartilaginous and osseous tissues. When performing this process, we observed several remarkable features of the method, which was discussed below.

To extract texture features, we introduced an image patch-based method which carries out feature vector computations after dividing each image into smaller size images. In order to capture the local morphologies of the cartilaginous and osseous tissues, the partition size of images was set to a 64 × 64 pixel image, which corresponds to an area of 16 × 16 μm^2^. This image size was large enough to capture the local structures, including a few lacunae, and hence may be a typical size for characterizing tissue morphology. From these images, we calculated the feature vectors using the GLCM method, which is a popularly used statistical texture analysis method. We demonstrated that the resulting parameters were sufficient to provide significant geometric information in characterizing the articular cartilage and subchondral bone tissues. For example, ASM values in Bone patches were lower than those in Cartilage patches, reflecting a relatively stronger intensity from the osseous components than the cartilaginous ones (Supplementary Fig. 2). Since the ECM of the subchondral bone consists primarily of type I collagen, which emits a strong SHG signal with thick fibrous morphology, while the main collagenous component of cartilage is type II collagen, which emits a relatively weaker signal and exhibits homogenous intensity profiles in the cartilage ECM. Furthermore, within Bone patches, ASM values at which the fibrous collagens exist were relatively lower, while within Cartilage patches, lacunae showed higher ASM values compared with the surrounding ECM, indicating that the heterogeneous collagenous structures are reflected in the ASM. Other texture parameters were also able to capture differences in the fibril structures in different interpretations. Thus, these results suggested that the geometric information based on image patches are suitable for characterization and discrimination of hyaline and fibrous collagen patterns emitted from the cartilaginous and osseous SHG signals. Living tissues and organs have particular structures, derived from the distribution of cells and ECM organization, which include a wide variety of local texture information depending on local tissue organization. Therefore, it is reasonable to describe image features by dividing the whole image into smaller patches, before evaluating them using the integrated statistics from texture parameters.

Once the feature vectors of the image patches were calculated, it was possible to apply these vectors to the BoF image classification analysis. We demonstrated the feasibility of performing classification of collagenous SHG morphology based on texture information. We showed that the classification accuracy of the texture-BoF algorithm is always higher than that of the SURF-BoF algorithm, where SURF is a major feature descriptor used here for comparison. One reason for this performance result may be that the GLCM includes information of the intensity profiles of the images, while the SURF descriptor is a gradient-based feature extractor. The intensity profiles contribute to the texture parameters as demonstrated by the fact that the osseous SHG images showed stronger intensity than the cartilaginous ones. Furthermore, we investigated how the BoF parameters influence the results. A remarkable point is that the codebook size independence only exists in the texture-BoF framework. The codebook size in the BoF framework is defined by how many vocabulary terms are constructed in the process of *k*-means clustering to create visual words. Generally, the codebook size takes on higher values such as *k* = 100–1000 within the BoF framework. The higher this value becomes, the higher the accuracy achieved. The SURF-BoF method inherited this tendency in contrast to the texture-BoF method. Although the reason why is not clear, this suggested that a large codebook size is not required, implying lower computational demand. In order to investigate how small a value of *k* is enough to classify the cartilaginous and osseous tissue SHG images, we tried to classify the images for *k* = 10 and *k* = 5. The computation of the *k* = 10 case still demonstrates a high accuracy of image classification results, but the accuracy for *k* = 5 case decreased ~60% (Supplementary Table 5).

A typical feature of the BoF method is an unordered collection of image patch information. This may be beneficial when analyzing living tissues because repetitive and periodic patterns are typically observed anywhere in many types of living tissues. In cartilage, the chondrocytes are distributed in almost equal distance with the neighboring cells, showing periodic distributive patterns. Therefore, applying the BoF method to other tissue collagen patterns and disease models will provide interesting future directions for study.

A weakness of the BoF method is computational costs due to the use of large numbers of image patches. As the number of patches per image increases, so does the classification accuracy, as we have demonstrated. We found the influence of different grid steps on the performance of the BoF framework to be consistent for the majority of the classification scenarios evaluated, and propose a high density grid as the preferred solution despite the higher computational demands.

The classification performance of the normal cartilage and bone tissues is high at ~90% accuracy. In contrast, the accuracy of fibrous cartilage classification is relatively low at 75%, depending on the codebook size and grid spacing. The areas where fibrillation occurred were found to be smaller than the area where the hyaline cartilage appears. Thus, the classifier recognized it as hyaline cartilage in many cases. To increase the classification accuracy, we need to increase the number of patches, alter the kinds of texture parameters, or change the patch extraction method. This would suggest that detecting the initial symptoms of OA and staging OA symptoms accurately requires improvements to the method, including spectral information in the descriptor, optimizing the number and types of images for codebook construction, and training. The use of autofluorescence and polarization dependence of excitation light is also a subject of future research.

Multi-photon excitation microscopy is becoming a widely used method to observe living, thick, and opaque tissues such as cartilage and bone in a non-invasive manner. Upon two-photon excitation, a strong SHG signal is generated specifically from collagen molecules. Therefore, changes in collagen architecture that occur in the cartilage matrix can be obtained by high-resolution and high-sensitivity imaging. Because the SHG does not require the use of any stains, approaches using this imaging technique to assess the degeneration of the cartilage matrix and its pathological states are increasingly being used to characterize OA pathology. It was demonstrated that morphological changes can be detected in degenerative bovine articular cartilage^12^, and in degenerative equine articular cartilage^13,38^. We have already studied the capabilities of SHG imaging for characterizing histopathological changes induced by OA^14^. Furthermore, in morphological investigations of early stage of human osteoarthritis, cartilage assessment was performed and clinical application was implied^15^. Additionally, miniaturized probes for multiphoton excitation microscopy were developed, which may be advantageous for overcoming constraints on the use of nonlinear microscopy in clinical studies^39^. Therefore, morphological studies of the articular cartilage by SHG microscopy can be a significant tool for assessing the OA disorder^16^. However, quantitative methods and a comprehensive strategy using advanced machine learning methods to assess the disease state of cartilage remain elusive. The texture feature-based classification method for SHG images presented in this paper could have clinical applications for detecting initial symptoms of OA. As a first step in the development of a method for implementing digital classification of the pathological state, here we have used the severe model of OA, which exhibits clear visual differences in the OA cartilage. Milder models that resemble the early stages of OA will be useful for automated detection of subtle but still distinct SHG patterning changes in cartilage.

Image texture analysis is a method which numerically represents features of complex visual patterns, including roughness, randomness, periodicity, and brightness. This method is starting to be used to analyze biomedical imaging data^18,20^. The GLCM texture analysis method has been used in MRI applications^22–24^, as well as on CT^25^, ultrasonic^26^, histopathological^27^, and cell images^28,29^. Focusing on the GLCM texture analysis applied to the SHG images, there are several examples in the literature of investigations of the utility of the computational method in characterizing cartilage ECM modifications under mechanical and biochemical constraints^40^, exploring the relation between collagen density in breast cancers and lymph node metastasis^41^, and evaluating collagen organization in skin dermis^42,43^. As for the purpose of the OA assessment, we have verified in this study that the GLCM pattern analysis allowed the discrimination of cartilaginous and osseous tissue in mouse joints. Within the BoF image classification framework applied to the OA cartilage pathology assessment, we demonstrated performance results higher than those implemented by the SURF descriptor. Therefore, unlike the generic image classification problem, the statistics-based texture features are more capable of discriminating biomedical images. Therefore, these results suggest that texture analysis is useful for detecting disease states, and its application to medical issues concerning the quantification of digital images increasingly becomes important.

## Materials and Methods

### Ethics statement

All animal experiments were approved by the Ethics Committee for Animal Experiments of Ehime University (#05-RE-2–16). The experimental procedures we employed were conducted in accordance with the approved guidelines.

### Surgery-induced OA model mice

Male C57BL/6 J mice were purchased (CLEA Japan, Inc.), and six-week old mice were subjected to surgery to produce an OA model. The surgical induction of the severe OA model was performed according to^37^. The knee joints of the mice were prepared (the bilateral hind limbs were shaved) for the surgery under general anesthesia with isoflurane. The patellar ligament, anterior/posterior cruciate ligaments and medial/lateral collateral ligaments were transected, and the medial/lateral menisci were removed using a stereomicroscope. The skin was closed after surgery. The contralateral knee joint was sham-operated without any ligament transection and meniscectomy to produce the control side model. The mice were sacrificed via cervical dislocation and their femurs harvested eight weeks after surgery. In total, twelve mice were subjected to surgery. The harvested femur samples were fixed overnight in 4% paraformaldehyde (PFA) diluted in phosphate-buffered saline (PBS) prior to multiphoton excitation microscopy and histological analyses.

### Preparation of tissue sections and SO staining

For preparations of tissue sections, excised femurs were fixed overnight in 4% PFA in PBS. The specimens were decalcified in 10% ethylenediaminetetraacetic acid (EDTA) at 4 °C for two weeks, and subsequently embedded in paraffin. Four-micrometer thick frontal sections were cut from the medial condyle of the joints. The sliced sections were then deparaffinized with xylene, and subjected to SHG imaging. After performing SHG imaging, the sections were stained with Safranin O Fast Green. Bright field images of the sections were acquired using a wide field inverted microscope (All-in-one fluorescence microscope BZ-X700, Keyence, Inc.) with a 20× magnification objective lens (PlanFluor 20× NA:0.45, Nikon). SHG images of the sections were acquired using multi-photon excitation microscope (A1R-MP, Nikon, Inc.) as described in detail below. For the sections, four mice samples were subjected to the analysis.

### SHG image acquisition

In order to perform SHG image acquisition, we utilized an upright multi-photon excitation microscope (A1R-MP, Nikon, Inc.). The microscope was equipped with a water immersion objective lens (CFI75 Apo 25 × W MP, NA:1.1, Nikon, Inc.) and a Ti:sapphire laser oscillator system (MaiTai eHP, Spectra-Physics, Inc.) with no additional optical modules for generating polarized light. To observe intact cartilage tissues, excised femurs were embedded in 1% agarose and the medial condyle was exposed under the objective lens of the microscopy system as previously described^14^. The images were acquired as *z*-stack image sequences with a step size of 3 μm ranging from the deepest portions to the surface of the cartilaginous tissue. To observe tissue sections, sections mounted on glass slides were placed on the microscope stage before acquiring *xy*-images. All SHG images were acquired at an excitation wavelength of 880 nm. To detect the SHG signal, we employed a dichroic mirror at 458 nm and an emission filter at 440/40 nm (center wavelength/bandwidth). The field of view of the acquired images was 0.5 mm × 0.5 mm and the resolution was 2048 × 2048 pixels, i.e. the pixel size was 0.25 μm. The images originally recorded as 12-bit gray level images were converted to 8-bit gray level images for GLCM computation.

### Image categorization

For the machine learning-based classification test, we prepared two kinds of image sets, namely a set of images acquired from tissue sections of normal articular cartilage and a second set from intact tissues of the OA model. To analyze the sections, the images acquired by the SHG microscopy (recorded as 2048 × 2048 pixel images) were trimmed to 512 × 512 pixel size sub-images so that the cropped images were confined almost exclusively to either the subchondral bone or cartilage regions. During this trimming process, the images were first examined as SO-stained sections. Subsequently, regions were selected and the cropped images were created. Thus, these images were categorized as either ‘Bone’ or ‘Cartilage’. During the analysis of tissue sections, four mouse samples were used and a total of 133 ‘Bone’ tissue images and 328 ‘Cartilage’ tissue images were obtained. For the analysis of *ex vivo* SHG images, *z*-stack image sequences were separated as individual *xy*-images before these images were divided into 4 × 4 blocks, each 512 × 512 pixels in size. To eliminate images in which most regions are empty with respect to the SHG signal, images possessing high signal levels were selected. The criterion used was that more than 25% of the image area was detected as signal region based on gray-level thresholding.

Our previous observation of the knee joints of the OA model successfully described the tissue types of hyaline cartilage, fibrous cartilage, and bone by SHG image patterns, that were consistently confirmed using well-established histological methods^14^. Therefore, to categorize the images obtained from OA model mice samples, we first examined the SHG images visually by comparing the SO-stained sections, and divided them manually into three distinct categories, namely ‘Hyaline’, ‘Fibrous’, and ‘Bone’. ‘Hyaline’ images were obtained from the control side of the SHG images, while ‘Fibrous’ and ‘Bone’ images were obtained from the OA side of the SHG images. ‘Hyaline’ images predominantly contain ordinary structures of cartilage, such as lacunas, chondrocytes and extracellular matrix. ‘Fibrous’ images show fibrotic SHG signals typically located around the surface of cartilage, reflecting the hyperplasia of fibrous cartilage. ‘Bone’ images display the collagenous structure of the subchondral bone, indicating that the bone tissue was exposed in OA model joints. In total, we obtained 2612 ‘Hyaline’ images, 102 ‘Fibrous’ images, and 301 ‘Bone’ images.

### Texture feature calculations

Image texture analysis is a mathematical method enumerating complex visual patterns of digital images, which reflect properties such as lightness, uniformity, density, roughness, regularity, linearity, frequency, and randomness^17–20^. Approaches to image texture analysis are categorized as either structural^44,45^, statistical^21,46^, model-based^47–49^, or transform^50^. In this study, we employed a statistical texture analysis method using the gray-level co-occurrence matrix (GLCM)^21^. Cartilaginous and osseous SHG images possess a large amount of texture information without any particular patterns. Therefore, a statistical approach seems to be suitable for our case. The GLCM approach is based on the use of second-order statistics of the gray scale image histogram. The GLCM is constructed by counting the number of occurrences of gray levels for pixel pairs, which are spaced apart from each other by distance *d* and angle *θ*. Each count is divided by the total count to obtain a probability. In this study, we chose *d* = 1 and *θ* = 0°, 45°, 90°, or 135°. The result is output as a matrix, denoted as *p*(*i, j*) where *i* and *j* are indices of rows and columns of the *N* × *N* matrix respectively, representing the probability of the gray-level co-occurrence pixels. From this matrix, several parameters can be calculated and image texture is characterized by these parameters. Here, we used eight texture parameters, correlation, angular second momentum (ASM), contrast, inverse difference moment (IDM), entropy, sum entropy, sum average, and sum variance, which are defined as

1 $${\rm{Correlation}}=\sum _{i,j}^{N}\,(i-{\mu }_{i})(j-{\mu }_{j})p(i,j)/{\sigma }_{i}{\sigma }_{j},$$

2 $${\rm{ASM}}=\sum _{i,j}^{N}{(p(i,j))}^{2},$$

3 $${\rm{Contrast}}=\sum _{i,j}^{N}{(i-j)}^{2}p(i,j),$$

4 $${\rm{IDM}}=\sum _{i,j}^{N}\frac{1}{1+|i-j|}p(i,j),$$

5 $${\rm{Entropy}}=\,\sum _{i,j}^{N}p(i,j)\mathrm{log}(p(i,j))\,,$$

6 $${\rm{Sum}}\,{\rm{entropy}}=\sum _{i}^{2N-2}{p}_{x+y}(i)\mathrm{log}({p}_{x+y}(i)),$$

7 $$\mathrm{Sum}\,\mathrm{average}=\sum _{i}^{2N-2}i\cdot {p}_{x+y}(i),$$

8 $${\rm{Sum}}\,{\rm{variance}}=\sum _{i}^{2N-2}{(i-\mathrm{Sum}\mathrm{average})}^{2}\cdot {p}_{x+y}(i),$$

where *μ* and σ are the mean and the standard deviation of the GLCM, respectively. The definition of *p*_x+y_(*i*) is given by $${p}_{x+y}(i)=\,\sum _{\begin{array}{c}j,k\\ j+k=i\end{array}}^{N}p(j,k)$$. Among them, ASM returns the sum of the squared elements of the GLCM, and gives information about the homogeneity of the image; homogeneous images are characterized by high ASM. ASM is highest in a constant image with a uniform gray-level and lower for those with more variation in images. Contrast indicates the variance between the gray levels of a pixel and its neighboring pixel. High contrast occurs when an image has a number of pixel pairs with large differences in gray levels. Correlation measures how correlated a pixel is to its neighboring pixel over the whole image. The more this parameter increased, the more linear the texture elements will appear. IDM is a measure of the homogeneity of the image. This feature takes the highest value (=1) for a constant image, and gives information on the similarity of a pixel’s value to those of its neighboring pixels in the image. In our analysis, the following strategy for calculating feature vectors was adapted to capture the characteristic structures of the osteochondral tissues. We used the regular square grid to represent the images as a collection of patches to be subjected to feature extraction (Fig. 2B). The grid steps were either 32 or 64 pixels, and the extracted image patch size was 64 × 64 pixels. To construct feature vectors for an image patch, we selected regions of interest (ROIs) composed of 8 × 8 blocks of 8 × 8 pixel images divided from a given patch. The GLCM calculation was performed individually on each ROI. After obtaining a set of GLCM parameters, the probability distributions of the parameters were computed to create feature vectors for an image patch (Fig. 2C). To avoid the influence of empty images on our analysis, we selected image patches with high signal levels over patches.

### Bag of Features computations

BoF has been proposed as a method for generic visual categorization, which is the problem of identifying various kinds of objects within images. This approach to image classification is based on an unordered collection of quantized image descriptors derived from local image patches, in which they discard any spatial information. In brief, the BoF represents a histogram of the number of occurrences of particular patterns in a given image. The BoF methods have been applied to image classification, object detection, and image retrieval. Due to its simplicity and performance, the BoF approach has been established in the field of computer vision. For a survey of relevant literature for BoF development and application, see^31^.

The BoF framework is illustrated schematically in Fig. 3. The first step in the BoF algorithm is to construct the codebook, which consists of quantized vectors in a feature space called visual words. For this, the feature vectors are extracted from the patch sets from each image and added to the codebook feature space. Next, to develop the codebook, the *k*-means clustering method with *k* = 20, 50, or 100 is performed. All the extracted feature vectors are thereby partitioned into *k* regions in which each feature vector belongs to the region with the nearest centroid. The extracted feature vectors are thus quantized to the centroid values in a codebook space, in which each cluster represents a visual word. Within the BoF framework, an image in the training and test image sets is represented as term vectors, which is a normalized histogram of the visual words. Given an image in the sets, features are detected and assigned to the nearest codes in the codebook. Here, we employed a *k*-nearest neighbor (kNN) classifier to assign the extracted features from images in a training set to the closest terms in the codebook. We went on to record the counts of each term that appears in the image to create a term vector. For machine learning, we used the SVM classifier for binary categorization of image sets of tissue sections and the multiclass error-correcting output codes (ECOC) classifier for ternary categorization of image sets of the OA model. For the image classification test, feature vectors are detected and assigned to their nearest matching terms from the codebook in a way used in the training stage, and eventually constructed a term vector. A test image is subsequently predicted to fall into one of the predefined categories.

Within the BoF machine learning framework, for computational simplicity, we used four texture parameters, correlation, ASM, contrast, and IDM. According to the feature vector construction scheme described above, we have a vector if we arrayed the histograms with twenty-two bins for different *θ*s in a line as $h=({h}_{\theta 1},{h}_{\theta 2},{h}_{\theta 3},{h}_{\theta 4})$, where ${\theta }_{1}=0^\circ ,\,{\theta }_{2}=45^\circ ,\,{\theta }_{3}=90^\circ \,{\rm{and}}\,{\theta }_{4}=135^\circ$. We thereby obtained a vector by arraying the vectors for four texture parameters in order, $V=({h}_{Cor},{h}_{ASM},{h}_{Con},{h}_{IDM})$. *V* therefore has the dimension of 352 and was used as a feature vector for an image patch.

In order to compare an existing feature extraction method with our texture analysis method, we used the speeded-up robust feature (SURF)^36^, which is a popular gradient-based feature description technique. The BoF parameters we have are the grid step *g* and codebook size *k*. The grid step controls sampling density, which is horizontal and vertical displacement of each feature center to the next. The codebook size, which corresponds to the dimensions of the term vectors, is configured during the clustering stage of the BoF algorithm. We investigated the parameter sensitivity of the classification method by varying these parameter values. In order for all images in a set to be tested, we employed the strategy of running several consecutive implementations of the BoF algorithm (Supplementary Tables 1 and 4). To analyze the tissue sections, one of four sets from each subset was tested in one run of the algorithm. All images were used for vocabulary building while the other images were used for training (Supplementary Table 1).

### Numerical calculations

All the calculations were performed using the software MATLAB (Mathworks Inc.).

### Data availability

The datasets generated during and/or analyzed during the current study are available from the corresponding author on reasonable request.

## Electronic supplementary material

Supplementary Information
