# Supplementary material for: Quantitative Morphometry for Osteochondral Tissues Using Second Harmonic Generation Microscopy and Image Texture Information
Source: Sci Rep. 2018 Feb 12;8:2826. doi: 10.1038/s41598-018-21005-9 (PMC5809560; doi:10.1038/s41598-018-21005-9)
Supplement: Supplementary file 1 — Supplementary Information [file 41598_2018_21005_MOESM1_ESM.pdf]

Quantitative Morphometry for Osteochondral Tissues Using Second Harmonic Generation Microscopy and Image Texture Information

Takashi Saitou[1,2,3], Hiroshi Kiyomatsu[4] and Takeshi Imamura[1,2,3]

[1] Department of Molecular Medicine for Pathogenesis, Graduate School of Medicine, Ehime University, Shitsukawa, Toon-city, Ehime 791-0295, Japan

[2] Translational Research Center, Ehime University Hospital, Shitsukawa, Toon-city, Ehime 791-0295, Japan

[3] Division of Bio-Imaging, Proteo-Science Center (PROS), Ehime University, Shitsukawa, Toon-city, Ehime 791-0295, Japan

[4] Department of Orthopedic Surgery, Graduate School of Medicine, Ehime University

Supplementary Information

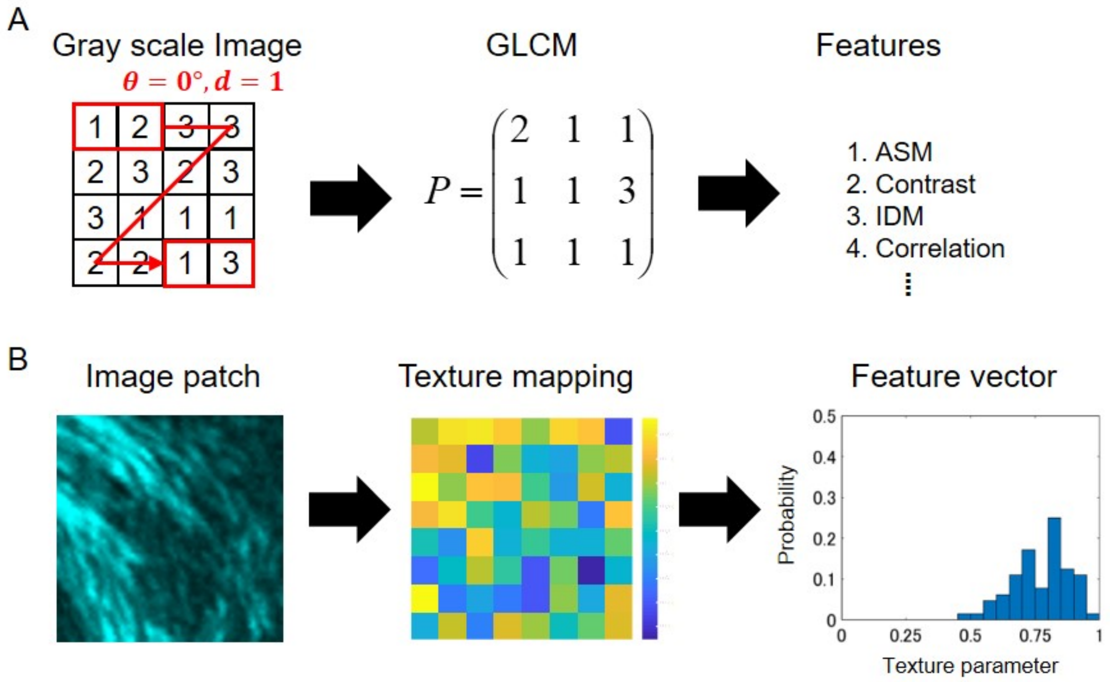

Supplementary Figure 1. Procedure for texture feature calculations. (A) The GLCM is calculated from a gray-level image by counting the number of occurrences of gray levels for pixel pairs, which are apart from each other with distance d and angle θ. This matrix is converted into several features. (B) For a given image patch, ROIs composed of 8 × 8 blocks of 8 × 8 pixel images are selected and each ROI is subjected to texture calculations. The calculated features are mapped on to the images, and the histogram of the GLCM features comprises the feature vector of the image patch.

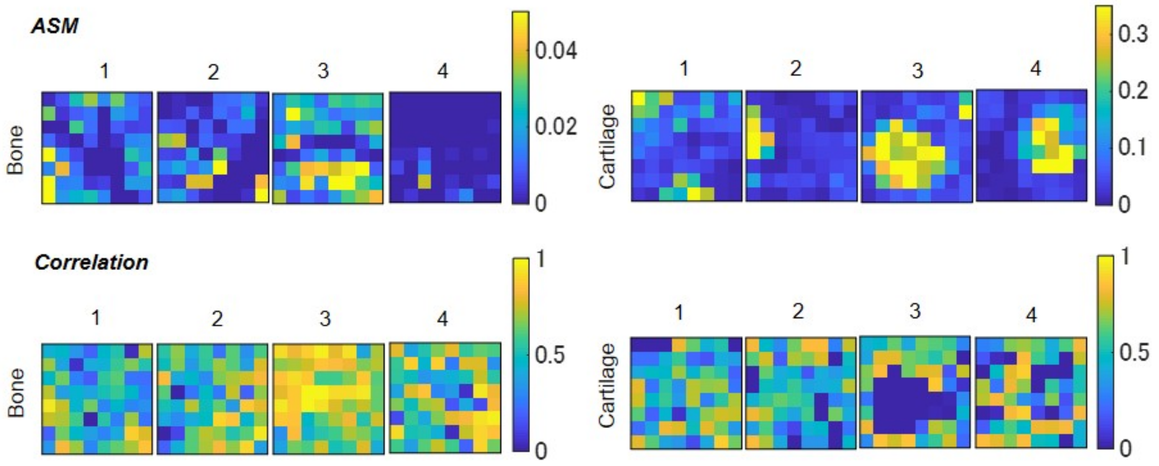

Supplementary Figure 2. Feature vector mappings for ASM and correlation corresponding to the images displayed in Figure 2B.

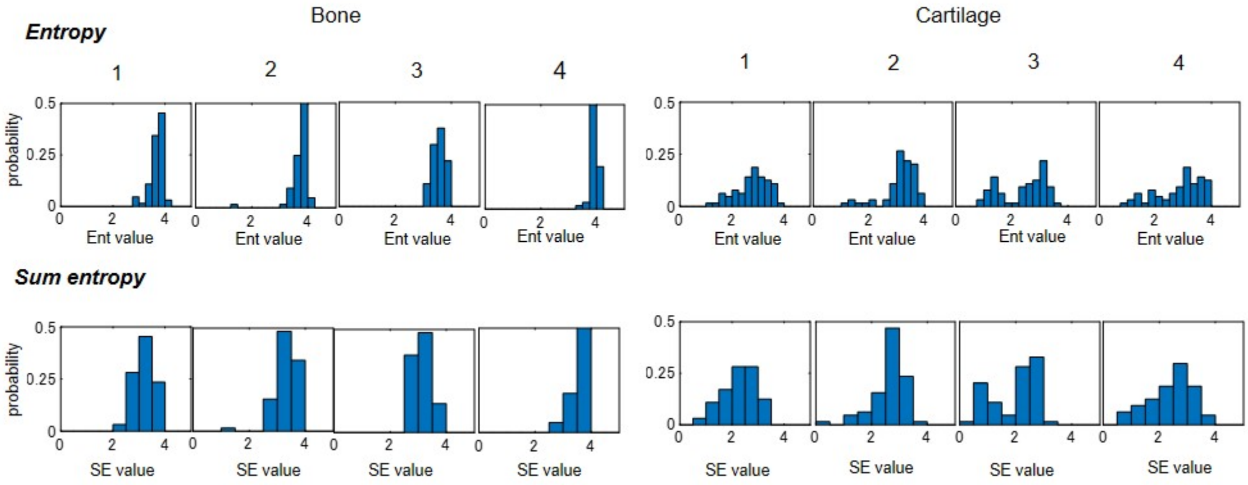

Supplementary Figure 3. Feature vectors of entropy and sum entropy corresponding to the images displayed in Figure 2B. Ent value: Entropy value. SE value: Sum entropy value.

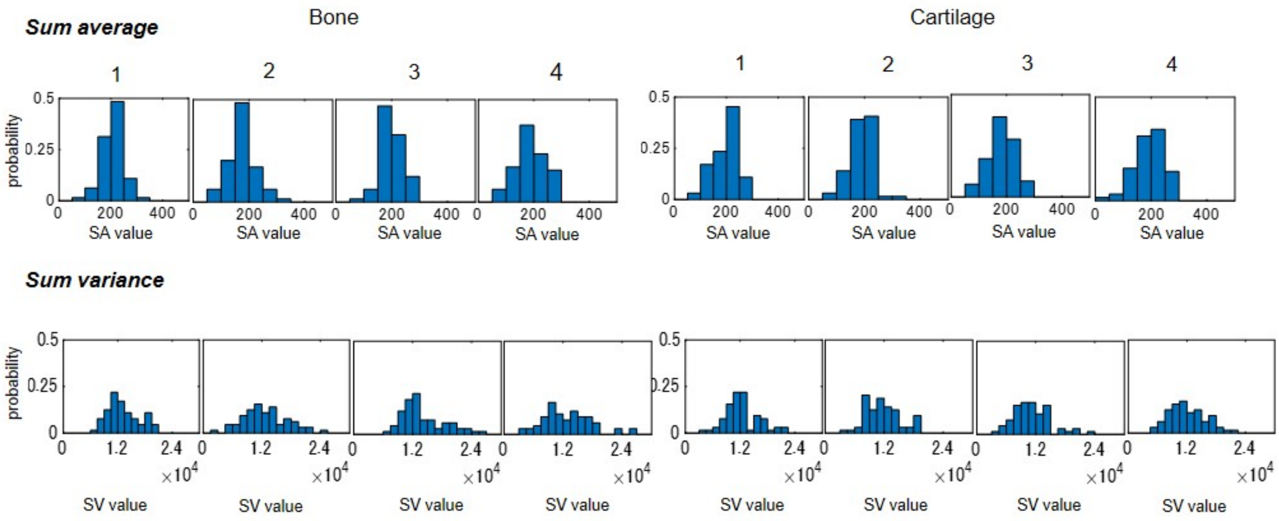

Supplementary Figure 4. Feature vectors of sum average and sum variance corresponding to the images displayed in Figure 2B. SA value: Sum average value. SV value: Sum variance value.

	Image set 1	Image set 2	Image set 3	Image set 4
Run #1	Test	Training	Training	Training
Run #2	Training	Test	Training	Training
Run #3	Training	Training	Test	Training
Run #4	Training	Training	Training	Test

Supplementary Table 1. Run strategy for classification tests performed on SHG images of tissue sections.

	Cartilage	Bone
$\boldsymbol{k = 20,\ g = 64}$		
Cartilage	0.755	0.245
Bone	0.215	0.785
$\boldsymbol{k = 50,\ g = 64}$		
Cartilage	0.864	0.136
Bone	0.061	0.939
$\boldsymbol{k = 100,\ g = 64}$		
Cartilage	0.889	0.111
Bone	0.072	0.928
$\boldsymbol{k = 50,\ g = 32}$		
Cartilage	0.883	0.117
Bone	0.044	0.956

Supplementary Table 2. Results of SURF-BoF classification tests performed on SHG images of tissue sections.

	Hyaline cartilage	Fibrous cartilage	Exposure of Subchondral bone
Number of mice	3	6	3

Supplementary Table 3. Classification of degenerative changes to cartilage in the OA model.

	Hyaline	Fibrous	Bone
$\boldsymbol{k = 20,\ g = 64}$			
Hyaline	0.754	0.116	0.130
Fibrous	0.341	0.278	0.381
Bone	0.124	0.075	0.803
$\boldsymbol{k = 50,\ g = 64}$			
Hyaline	0.830	0.101	0.070
Fibrous	0.299	0.326	0.375
Bone	0.059	0.138	0.803
$\boldsymbol{k = 100,\ g = 64}$			
Hyaline	0.830	0.093	0.077
Fibrous	0.271	0.412	0.317
Bone	0.071	0.159	0.770
$\boldsymbol{k = 50,\ g = 32}$			
Hyaline	0.875	0.079	0.045
Fibrous	0.277	0.414	0.310
Bone	0.034	0.102	0.864

Supplementary Table 4. Results of the SURF-BoF classification tests performed on SHG images of the OA model.

	Cartilage	Bone
$\boldsymbol{k = 10,\ g = 64}$		
Cartilage	0.970	0.029
Bone	0.150	0.850
$\boldsymbol{k = 5,\ g = 64}$		
Cartilage	0.608	0.392
Bone	0.404	0.596

Supplementary Table 5. Results of the texture-BoF classification tests for smaller *k* values performed on SHG images of tissue sections.
